# Contextual analysis for the implementation of a digital psychosocial intervention to enhance mental health outcomes in North Macedonia

**DOI:** 10.1192/j.eurpsy.2025.1438

**Published:** 2025-08-26

**Authors:** M. Milutinovic, L. Novotni, A. Novotni, S. Bajraktarov, S. Arsova

**Affiliations:** 1 University Clinic of Psychiatry, Skopje, North Macedonia

## Abstract

**Introduction:**

This study represents the first effort in North Macedonia to examine the contextual attributes that may influence the effectiveness and acceptability of a novel digital intervention, DIALOG+, within the mental health care system. The intervention aims to enhance mental health outcomes through a structured approach, but its success depends on understanding the specific characteristics of the local health context.

**Objectives:**

The primary objective of this research is to identify the key contextual attributes within the mental health care system of North Macedonia that are relevant to the successful implementation of DIALOG+. This includes examining factors that could impact both the effectiveness of the intervention and its acceptance by various stakeholders, including patients, clinicians, carers, and policymakers.

**Methods:**

Data for this study were drawn from a variety of sources, including the National Mental Health Strategy 2018-2025, relevant documents from the World Health Organization, and other action plans. In addition, interviews were conducted with key stakeholders—patients, carers, clinicians, and policymakers—to gather perspectives on the anticipated introduction of DIALOG+ and assess the readiness of the mental health centers for its implementation. The data were subsequently mapped to a framework developed by the Ottawa Implementation Group, which outlines 14 key contextual attributes influencing health interventions.

**Results:**

The findings were categorized into two subgroups, identifying both facilitators and barriers to the implementation of DIALOG+ in North Macedonia’s mental health system. The intervention’s characteristics as a broadly applicable psychosocial tool align well with modern approaches to psychosocial rehabilitation, particularly for individuals diagnosed with psychosis.

**Conclusions:**

DIALOG+ presents a valuable tool for mental health professionals in North Macedonia, offering structured support for monitoring patient progress and achieving institutional objectives. The intervention has the potential to facilitate patients’ reintegration into society, enhance their independence, and enable them to reach their full potential in the pursuit of a healthy and functional life.

**Disclosure of Interest:**

None Declared

